# Association of health literacy and cognition levels with severity of adverse drug reactions in cancer patients: a South Asian experience

**DOI:** 10.1007/s11096-020-01062-9

**Published:** 2020-05-30

**Authors:** Vishal Gupta, Gangachannaiah Shivaprakash, Dipanjan Bhattacherjee, Karthik Udupa, Basavaraj Poojar, Ravi Sori, Shubhangi Mishra

**Affiliations:** 1 Department of Pharmacology, Manipal Academy of Higher Education, Manipal, Karnataka India; 2 Department of Medical Oncology, Manipal Academy of Higher Education, Manipal, Karnataka India

**Keywords:** Adverse drug reactions, Chemotherapy, Cognition, Health literacy, India

## Abstract

*Background* The occurrence of adverse drug reactions with chemotherapy among cancer patients is a well-documented phenomenon. However, the understanding of contributoring factors and their influence on the severity of adverse drug reactions is incomplete without the psychosocial factors affecting them. *Objective* The present study was done to understand if factors like Health literacy and cognition levels have an association with the severity of adverse drug reactions of cancer chemotherapy. *Setting* This study was done in the Department of Medical Oncology in a tertiary care hospital in India. *Method* Two hundred and twenty-four patients meeting the study inclusion and exclusion criteria took part in the study. Details of adverse drug reactions were collected as per the central drugs standard control organization format and severity of adverse drug reactions assessed with National Cancer Institute common terminology criteria of adverse events, version 5.0. Health Literacy and Cognition Levels of patients were assessed using standardized questionnaires, i.e., Short test of functional health literacy in adults and short portable mental status questionnaire, respectively. Data were anonymized and analyzed using Statistical Package for Social Sciences version 16.0 software. Pearson’s Chi square test (*p* value **≤ **0.05 was considered statistically significant) was used to study the associations. *Main outcome measure* The associations of Health Literacy and Cognition Levels with the severity of adverse drug reactions. *Result* We found that both Health Literacy and Cognition Levels had a statistically significant association with Grade 3 and above adverse drug reactions in cancer patients receiving chemotherapy. *Conclusion* An initial assessment of Health Literacy and Cognition Levels in cancer patients by cancer care providers can help identify patients at high risk of developing severe adverse drug reactions. Interventional measures for improving Health Literacy by healthcare providers can help reduce the overall burden of disease on the patient due to adverse drug reactions.

**Electronic supplementary material:**

The online version of this article (10.1007/s11096-020-01062-9) contains supplementary material, which is available to authorized users.

## Impacts on practice

- An initial assessment of Health Literacy and Cognition Levels may help identify oncology patients at risk of developing severe Adverse Drug Reactions.
- Less severe Adverse Drug Reactions may reduce the global healthcare costs which may act as an incentive to cancer care providers at improving Health Literacy.
- Early recognition and reduced severity of Adverse Drug Reactions due to cancer chemotherapy may also improve health-related quality of life in cancer survivors.

## Introduction

Functional Health Literacy (HL) and formal education are two separate entities. Clinicians often consider an uneducated patient to be illiterate in health behavior also whereas an educated patient may not necessarily be health literate too. The World Health Organization (WHO) defines HL as the cognitive and social skills which determine the motivation and ability of individuals to gain access to, understand and use information in ways which promote and maintain good health [1].

Therefore, a highly educated person may not have an equal amount of understanding of health behavior too. In the National Adult Literacy Survey by Kirsch et al. [2], 44% of adult men were found to be literate but had inadequate functional HL. It implies that reading labels on medicine bottles, understanding doctor’s advice and prescription, and following medical instructions are not carried out in the expected form. As William et al. reported in two separate studies (done on patients with asthma and patients with hypertension and diabetes), individuals with inadequate HL are unable to take appropriate care of themselves despite education through special classes [3, 4].

The study of HL in different scenarios like in chronic illnesses, in older adults, has shown a high prevalence of inadequate HL. The outcome of this is that in the event of a medical complication or an adverse drug event, patients may not report to the hospital at the right time, thereby making their own management difficult and increasing the burden on medical fraternity and healthcare infrastructure. HL as an entity is more important in functioning as a patient with cancer because interactions with varied specialties for surgery, radiation, cycles of chemotherapy, and post-treatment care are complex [5]. Corroborating with this, Cartwright et al. [6] also concluded in their study that HL is an independent predictor of hospital admissions in cancer patients. Another meta-analysis of Adverse Drug Reactions (ADRs) in hospitalized patients by Lazarou et al. [7] found anti-cancer drugs to be the main contributor to deaths related to ADRs. In the context of older adults, specifically, Whittaker et al. [8] found Limited HL to be a serious risk factor for adverse events in general.

From the European subcontinent, Sørensen et al. did a survey in countries of Europe: Austria, Bulgaria, Greece, Germany, Ireland, the Netherlands, Poland, and Spain. This European health literacy survey (HLS-EU) done in 2015 showed that limited HL is an important challenge for health practices and policies across Europe, although to a different level in different countries [9]. Quaglio et al. [10] stated that almost 50% population in Europe is deficient in HL and proposed that European Commission and European Union Member States take necessary actions to increase HL at the individual, organizational, community, and national levels. Inadequate HL is also associated with poorer decision making as exemplified by Busch et al. [5] in their study on Colorectal cancer patients (CanCORS study). Song et al. [11] showed that poor HL was also associated with poor health-related quality of life in newly diagnosed patients of localized prostate cancer.

Along with HL, another psychosocial factor, i.e., the patient’s Cognition Level (CL), also plays an important role in the decision making and execution behavior of patients. Cognition is defined as any form of information processing, mental operation, or intellectual activity such as thinking, reasoning, remembering, imagining, or learning [12] patient’s memory and executive function are seen to affect health behaviors in a study done on Korean adults [13].

## Aim of the study

The present study proposed to examine the association between HL and CL on the severity of ADRs. We hypothesized that patients with inadequate HL are likely to have more severe ADRs because they are likely to present late. Similarly, low levels of cognition may also result in more severe ADRs.

## Ethics approval

The Institutional Ethics Committee approved all aspects of the present observational study (IEC No. 95/2015). The study was conducted in accordance with the Declaration of Helinski.

## Method

### Study design, study population and data collection

The present observational study was conducted prospectively over 6 months. The study subjects were cancer patients receiving chemotherapy in the Department of Medical Oncology in a tertiary referral center in Southern India.

As per the pharmacovigilance register maintained in the department of medical oncology, about 30% patients receiving chemotherapy were reported to have ADRs. At 20% relative precision and with a 95% confidence interval, the sample size was estimated to be 224 patients with ADRs. Adult patients more than 18 years of age, who were receiving cancer chemotherapy and were able to read the local language, were included in the present study. Self-reported illiterate patients, patients with dementia, or receiving treatment with drugs that may lead to cognitive impairment and patients with visual impairment, which is uncorrectable by glasses, were excluded from this study. Demographic parameters of the study subjects like age, sex, self-reported education were recorded. Additional data about the type of cancer, the type of chemotherapy regimen received, and the drugs implicated for ADRs were obtained from the medical records.

#### Identification and profiling of ADRs

The suspected ADR was identified by the trained study investigator, and details of ADRs were recorded as per the CDSCO ADR reporting form [14]. This form covers all vital information with respect to ADR like a brief description of the reaction, date of onset, outcome, the seriousness of the reaction, and recovery, along with the patient’s medical and drug history were recorded. The causality relationship of the ADR was established using the Naranjo scale as doubtful, possible, probable, and definite [15]. ADRs with the causality of possible, probable, and definite were included in the present study, and doubtful ones were excluded. Thereafter, the grading of the severity of ADRs was done as per CTCAE grading [16], and ADRs were recorded as Grade 1, Grade 2, and Grade 3 and above (for Grade 3, Grade 4 and Grade 5 ADRs). ADRs were also classified as serious or non-serious ones, if recovered completely or not, predictable or unpredictable, and whether preventable or non-preventable.

#### Measurement of health literacy (HL)

Functional HL of study participants was assessed by Short Test of Functional Health Literacy in adults (STOFHLA) [17]. It comprised of 36 different items in two passages with a proposed test duration of 7 min. The trained study investigator would read out a scripted introduction and instructions to the patient. Thereafter, the questionnaire was taken from the patient at the end of 7 min, irrespective of whether it had been completed. The scoring was done out of a total of 36, and functional HL was categorized as Inadequate HL (score 0–16), Marginal HL (score 17–22), and Adequate HL (score 23–36).

#### Measurement of cognition levels (CL)

Cognition levels of the patients was assessed by using Pfeiffer’s Short portable mental status questionnaire (SPMSQ) [18]. It comprised of 10 items to assess orientation, memory function, and capacity to perform mental operations. The questions were asked by the trained study investigator, and responses by the patients were recorded. The questionnaire was then analyzed based upon the number of errors done by the patient, cognition levels were graded as intact cognition (0–2 errors), mild impairment of cognition (3–4 errors), moderate impairment of cognition (5–7 errors), and severe impairment of cognition (8–10 errors).

Both the study questionnaires were translated into the local language, where the study was conducted.

#### Validation of translated questionnaires

The questionnaires in English were first translated into local language *kannada*. Experts from the department of public health and oncology verified the questionnaires by forward translation and backward translation. The translated questionnaires were administered to a pilot group of twenty subjects. Statements with unanswered responses and missed answers were rectified by the subject expert again. The statistician performed the content and construct validity. The final translated questionnaires were given to study investigators to be administered to the study subjects.

#### Statistical analysis/data analysis

Continuous variables were summarized using mean and SD. Variable which are categorical in nature, were summarized using frequency and percentages. Chi square test was used to investigate the significant association of ADR severity with the cognition levels and HL. A *p* **≤ **0.05 was considered to be statistically significant throughout.

## Results

### Demographic pattern

A total of 224 patients were included in the present study. 45.1% were males, and 54.9% were females. The mean age of the study population was 48.37 years, and 51 (22.7%) patients were more than 60 years of age. The types of cancer were breast cancer (22.7%), Non-Hodgkin’s lymphoma (12.2%), ovarian carcinoma (11.6%), leukemia (9.3%), lung cancer (7.1%) and other malignancies (37.1%). The most commonly implicated drug for ADRs in the present study was paclitaxel (15.2%), followed by doxorubicin (12.9%) and cyclophosphamide (12.5%).

### ADRs profile

ADRs were recorded for all 224 patients and categorized by their types as hematologic (33%), gastrointestinal (28.6%), neurological (3.5%), genitourinary (1.3%), respiratory (0.8%), dermatologic (4.5%), infective (5.3%), metabolic (1.7%) and others (21.3%). The causality as per the Naranjo scale showed 0.5% definite, 32.1% probable, and 67.4% possible ADRs. CTCAE grading of severity of ADRs showed 25.9% Grade 1, 33.5% grade 2, 40.6% grade 3 and above ADRs. Table 1 shows participant characteristics, and details of the ADRs recorded.

**Table 1:** Participant characteristics and ADR profiles

Characteristic	*n* (%)
Mean age (in years)	48.37
*Sex*	
Male	101 (45.1)
Female	123 (54.9)
*Education (self*-*reported)*	
School only	142 (63.4)
College Graduate	82 (36.6)
*Type of cancer*	
Breast cancer	51 (22.7%)
Non-Hodgkin’s lymphoma	27 (12.2%)
Ovarian carcinoma	26 (11.6%)
Leukemia	21 (9.3%)
Lung cancer	16 (7.1%)
Others	83 (37.1%)
*Seriousness of ADRs*	
Serious	129 (57.6)
Non-serious	95 (42.4)
*Outcomes of ADRs*	
Recovered completely	174 (77.7)
Others	50 (22.3)
*Predictability of ADRs*	
Predictable	203 (90.6)
Unpredictable	21 (9.4)
*Preventability of ADRs*	
Preventable	141 (62.9)
Nonpreventable	83 (37.1)
*Types of ADRs*	
Hematologic	74 (33)
Gastrointestinal	64 (28.6)
Neurological	8 (3.5)
Genitourinary	3 (1.3)
Respiratory	2 (0.8)
Dermatologic	10 (4.5)
Infective	12 (5.3)
Metabolic	4 (1.7)
Others	38 (21.3)
*Causality of ADRs*	
Probable	72 (32.1)
Possible	151 (67.4)
Definite	1 (0.5)
*Severity of ADRs*	
Grade 1	58 (25.9)
Grade 2	75 (33.5)
Grade 3 and above	91 (40.6)

Amongst the 91 patients with Grade 3 and above ADRs, 55 (60.4%) were females, and 36 (39.6%) were males. There were 21 patients of age more than 60 years who had Grade 3 and above ADRs. Complete recovery occurred in 72 (79.1%) of Grade 3 and above ADRs. More than 50% of Grade 3 and above ADRs were of hematologic type.

#### Association of HL and severity of ADRs

A cross-tabulation analysis by Pearson’s Chi square test showed a statistically significant association between inadequate HL and Grade 3 and above ADRs (Table 2). 47.8% had inadequate, 27.2% had marginal, and 25% had adequate HL.

**Table 2:** Association between health literacy and Grade 3 and above ADRs

Health literacy	Grade 1 ADR (58)	Grade 2 ADR (75)	Grade 3 and above ADRs (91)
Inadequate (107)	16	17	74
Marginal (61)	19	27	15
Adequate (56)	23	31	2

(*p *< 0.001 for Inadequate HL and Grade 3 and above ADRs)

#### Association between Cognition levels and severity of ADRs

Cross-tabulation analysis by Pearson’s Chi square analysis showed a statistically significant association between severe impairment of cognition levels and Grade 3 and above ADRs (*p* = 0.001, Table 3). 42.9% had intact cognition, 35.7% had mild impairment, 8.9% had moderate impairment, and 12.5% had severe impairment of cognition levels.

**Table 3:** Association between cognition levels and Grade 3 and above ADRs

Cognition levels	Grade 1 ADR (58)	Grade 2 ADR (75)	Grade 3 and above ADRs (91)
Intact (96)	32	39	25
Mild impairment (80)	18	24	38
Moderate impairment (20)	7	6	7
Severe impairment (28)	1	6	21

(*p *< 0.001 for severe impairment of cognition and Grade 3 and above ADRs)

## Discussion

The Global cancer statistics 2018 estimates the prevalence of cancer worldwide as 18.1 million newly diagnosed cases [19]. Dhillon et al. [20] declared that 8.3% of total deaths in India were due to cancer in 2016. Managing cancer is a high-cost task due to costs related to the treatment as well as the poor prognosis most of the time. The concurrent chemotherapy given to patients is well known to cause ADRs, which itself is a contributor to healthcare costs. In their study on a regional Cancer institute, Couffignal et al. [21] found that ADRs due to anti-cancer drugs contributed to 6.7% of the total hospital budget.

In addition to the financial burden associated with chemotherapy-induced ADRs, cancer care also includes surgical, restorative, rehabilitative, and psychotherapeutic interventions. This multi-faceted approach requires interactions between the patient and the healthcare provider at various levels. Therefore, HL and Cognition levels may play an important role in decision making when they interact with health care providers.

Various other aspects of the occurrence and the severity of these ADRs have been examined by Belachew et al. [22] in their study from Ethiopia that age, polychemotherapy, and the dose of the chemotherapeutic agent given, all had a significant association with Grade 3 and above ADRs.

The role of psychosocial factors like HL and CL remains much unexplored, to the best of our knowledge, in the context of the severity of ADRs. In a study by Theodore et al. [23] in COPD patients, it was found that lower HL is associated with poorer health status and outcomes. The evaluation of HL in chronic illnesses has been multipronged, but similar evaluation is needed in patients living with cancer. It is equally important to emphasize here that health literacy and formal education are separate and distinct entities.

As per Halverson et al. [24], patients with low HL have difficulty navigating a complex health care system, which translates into either underutilized preventive care or over-utilized emergency care.

Another important but worrying finding of this study was that 63% of ADRs were found to be preventable, which is in contrast to a previous study by Chopra et al. [25] who found that 49% of ADRs were either probably or definitely preventable. This could be due to the differential demographic profile and type of cancer in the populations studied.

Similarly, intact cognition impacts decision-making behavior as revealed by studies on patients with chronic illnesses [26]. Cognitive impairment and its effects have been studied in cancer subjects [27] as well as non-cancer subjects [13], but the impaired cognition levels affecting the severity of ADRs in cancer patients has not been examined so far.

Chemotherapy-induced cognitive impairment (CICI) is a known entity that occurs in cancer patients on chemotherapy [27]. The present study found that moderate to severe impairment of cognition levels was associated with grade 3 and above ADRs. The significance of such a finding lies in the fact that the assessment of cognition by cancer care providers at the inception stage of chemotherapy may help them in anticipating the pattern of the severity of ADRs. Also, it will help the patient and their caregivers at home to have realistic expectations from the treatment.

Improved cancer survivorship due to availability of better drugs and more efficient treatment regimens makes an initial assessment of factors like HL and CL as wise forethoughts to improve cancer-care related outcomes. Health-related quality of life is also an important marker in cancer prognosis and highly depends upon HL [24]. The International Union for Health Promotion and Education (IUHPE) position statement on HL emphasizes the global need to address the development of HL in all forms, i.e., by caregivers, healthcare providers as well as the healthcare system also. This cumulative approach can be used to design public policies and health interventions for global health advancement [18]. The study by Whittaker et al. demonstrated that educational interventions like interactive games were successful in increasing knowledge of older adults about medication safety and poison prevention when compared to handing over of a brochure of side effects [8]. In their study on patients of carcinoma prostate, Song et al. [11] proposed that although education of a person can serve as a proxy for HL in a clinical setup, however, for research purposes, a formal assessment of HL is needed. Quaglio et al. recommended that HL should be accelerated at both individual as well as practitioner level. They suggested improving digital skills at the patient level and development of clear local and national policies to improve HL at the practitioner level [10].

## Strengths and limitations

The present study is a novel concept, unexamined before, to the best of our knowledge, and gives a new dimension to consider inexpensive interventions like promoting HL to reduce the economic burden of serious ADRs. A sound HL environment in a healthcare institute acts as a backup to the efficient delivery of quality cancer care [24].

One of the limitations noted in the present study was that the sample size was not very large due to time constraints. As the study was conducted in a local Oncology department in Southern India, it restricted the representation of cancer patients from other demographic locations. Future multicentric studies need to be performed to arrive at more data concerning HL and CL in cancer patients experiencing ADRs. Responses to the questionnaires were based on self-report; therefore they were subject to recall bias and non-response.

Another limitation noted in this study was that the actual prevalence of inadequate HL might be higher in society as the self-reporting illiterate patients were excluded from the present study.

Assessment of HL includes the capacity to obtain, process, and understand basic health-related information, and S-TOHFLA captures more of reading comprehension and is of limited value in measuring numeracy.

## Future implications

The present study has suggested that patients with lower HL and CL are more vulnerable to experience more serious ADRs due to cancer chemotherapy; therefore a mandatory pre-assessment of HL and CL as a part of comprehensive cancer care can help identify patients at risk of serious ADRs. Treating oncologists can wisely choose therapeutic options, thereby avoiding highly toxic chemotherapeutic agents in patients with lower HL and CL.

Future multi-country studies can be performed to arrive at more conclusive data, which is representative of geographical areas from Europe, USA, and South-Asian countries.

## Conclusion

We concluded that lower HL and CL are associated with more severe ADRs in cancer patients on chemotherapy. This study has amplified the preexisting significance of HL in that an initial assessment must be a mandatory part of the planning of cancer treatment to predict the severity of associated ADRs. In addition to this, a parallel initial assessment of CL also can help by marking a preexistent impairment in cognition or any deterioration of it further after the initiation of chemotherapy. These recommendations can help reduce the overall financial burden on the health economy due to ADRs. Another recommendation from this study is to conduct future analyses on non-cancer patients to extrapolate these findings on patients with chronic illnesses like diabetes and hypertension.

## Electronic supplementary material

Below is the link to the electronic supplementary material.

Supplementary material 1 (XLSX 28 kb)
